# Raw or Pasteurized Milk

**Published:** 1916-07

**Authors:** 


### Raw or Pasteurized Milk.

Some years ago, a physician who was an alderman of the city of Buffalo, introduced an ordinance requiring all milk sold to be pasteurized. At a public hearing, several prominent physicians spoke against the measure, insisting on the superiority of clean, raw milk. In the last few years, there has been an increasing tendency toward the voluntary or compulsory pasteurization of milk, voluntary on the part of milk companies both as to ''talking point'' for their product and because pasteurization lessens the perishability of milk and renders its handling on a large scale more economic. In general terms, the pros and cons of raw and pasteurized milk have not changed. There is an obvious advantage in delaying decomposition and in lessening the bacterial content. On the other hand, it is still admitted by most authorities, with more or less scientific exactness of explanation, that pasteurization changes the constituents of milk so that, on the whole, it is not quite so valuable as a food as in the raw state. We prefer to leave the issue in these very general terms, to express no opinion as to the aggregate superiority of raw and pasteurized milk under such practical conditions as must apply to the large bulk of milk sold in large cities but to emphasize the fact that the time is ripe for a thorough, scientific and practical investigation of the matter, so that a policy so important to the public health may be firmly established without delay.

